# A novel tumor-progressing fibroblast signature derived from single-cell RNA sequencing enables prognostic stratification and reveals RNF11 as a functional regulator in bladder cancer

**DOI:** 10.3389/fmolb.2025.1722809

**Published:** 2026-01-15

**Authors:** Miaoyu Zhang, Liangbin Lin, He Xu, Xu Su, Kui Zeng, Fangyang Zhong, Xianchao Su, Jie Chen, Min Cao, Hui Yu, Hui Zhuo

**Affiliations:** 1 Department of Urology, The Third People’s Hospital of Chengdu, The Affiliated Hospital of Southwest Jiaotong University, College of Medicine, Southwest Jiaotong University, Chengdu, Sichuan, China; 2 Medical Research Center, The Affiliated Hospital of Southwest Jiaotong University, The Third People’s Hospital of Chengdu, Chengdu, China; 3 Obesity and Metabolism Medicine-Engineering Integration Laboratory, Department of General Surgery, The Third People’s Hospital of Chengdu, The Affiliated Hospital of Southwest Jiaotong University, Chengdu, Sichuan, China; 4 The Center of Gastrointestinal and Minimally Invasive Surgery, Department of General Surgery, The Third People’s Hospital of Chengdu, The Affiliated Hospital of Southwest Jiaotong University, Chengdu, Sichuan, China; 5 Department of Urology, The Affiliated Hospital of Southwest Jiaotong University, The Third People’s Hospital of Chengdu, Chengdu, Sichuan, China

**Keywords:** bladder cancer, cancer-associated fibroblasts, prognostic model, RNF11, single-cell RNA sequence

## Abstract

**Background:**

Cancer-associated fibroblasts (CAFs) are key drivers of tumor progression in bladder cancer (BLCA), yet their molecular heterogeneity and prognostic utility remain incompletely characterized. Single-cell studies have revealed distinct CAF subpopulations with divergent clinical impacts, necessitating refined prognostic frameworks that capture CAF-mediated progression.

**Methods:**

We analyzed single-cell RNA sequencing data (GSE267718) from 8 BLCA patients to identify CAF populations and define progression-associated gene signatures. Using 359 TCGA-BLCA samples as the training cohort, we performed non-negative matrix factorization (NMF) consensus clustering on 85 prognostically significant CAF genes, identifying two molecular clusters with distinct survival outcomes. Through LASSO-Cox regression and stepwise selection, we constructed a four-gene Tumor-Progressing Fibroblast Riskscore model comprising *FOXA1*, *TBX3*, *LRIG1*, and *RNF11*. Model performance was validated in the E-MTAB-4321 cohort (n = 476). Functional validation of *RNF11* was performed using shRNA-mediated knockdown in T24 and 5637 bladder cancer cell lines, followed by proliferation, migration, invasion assays, and transcriptomic profiling.

**Results:**

Single-cell analysis identified 557 differentially expressed genes between non-muscle-invasive bladder cancer and muscle-invasive bladder cancer CAFs. NMF clustering stratified TCGA patients into 2 clusters with significantly different overall survival. The TPFR model showed consistent prognostic performance in both training and validation cohorts, with high-risk patients showing significantly worse survival. Functional enrichment analysis revealed that TPFR scores correlated with ECM-receptor interaction, focal adhesion, and cytoskeletal regulation pathways. Stratified analysis revealed superior model performance in elderly (>60 years), male, and early-stage patients. In particular, *RNF11* knockdown significantly reduced proliferation, migration, and invasion in 5637 and T24 cells, while transcriptomic analysis revealed alterations in tumors after *RNF11* knockdown including TNF and MAPK signaling pathway, indicating a potential mechanism by which RNF11 regulates bladder cancer progression.

**Conclusion:**

We established a CAF-based prognostic model that integrates single-cell insights with bulk transcriptomics for robust risk stratification in BLCA. The TPFR model shows clinical utility particularly in elderly and early-stage patients. Functional characterization showed that RNF11 regulates proliferation and migration of bladder cancer. These findings highlight the prognostic value of CAF signatures and provide a framework for precision medicine approaches in bladder cancer management.

## Introduction

Bladder cancer (BLCA) is one of the top 10 most common malignancies worldwide, with approximately 573,000 new cases and 213,000 deaths annually (Sung et al., 2021; Bray et al., 2024). Based on the depth of tumor invasion, bladder cancer can be classified into non-muscle-invasive bladder cancer (NMIBC) and muscle-invasive bladder cancer (MIBC). NMIBC is characterized by tumors confined to the mucosa or submucosa layers, while MIBC denotes tumors that have invaded into or beyond the detrusor muscle layer (Lopez-Beltran et al., 2024). Despite advances in diagnosis and treatment, MIBC remains associated with poor prognosis, with 5-year survival rates ranging from 60%–70% (Patel et al., 2020). BLCA exhibits substantial histological heterogeneity, with urothelial carcinoma accounting for over 90% of cases, while the remaining cases comprise squamous cell carcinoma, adenocarcinoma, neuroendocrine tumors, and other rare histological subtypes (Tran et al., 2020; van Hoogstraten et al., 2023). The heterogeneous nature of BLCA, coupled with its high recurrence rate and progression risk, underscores the urgent need for robust molecular stratification systems to guide clinical decision-making (Kamoun et al., 2019).

The tumor microenvironment (TME) has emerged as a critical determinant of cancer progression and therapeutic response (Hanahan and Weinberg, 2011). Among TME components, cancer-associated fibroblasts (CAFs) represent the most abundant stromal cell type and play pivotal roles in tumor progression through extracellular matrix (ECM) remodeling, secretion of growth factors and cytokines, and modulation of immune responses (Chen and Song, 2019; Sahai et al., 2020). Recent single-cell RNA sequencing (scRNA-seq) studies have revealed substantial heterogeneity within CAF populations, identifying distinct functional subtypes including inflammatory CAFs (iCAFs), myofibroblastic CAFs (myCAFs), and antigen-presenting CAFs (apCAFs) (Biffi and Tuveson, 2020; Elyada et al., 2019). In BLCA, CAF-associated TGF-β activity has been mechanistically linked to T-cell exclusion and primary resistance to anti-PD-L1 in the IMvigor210 cohort, where a pan-fibroblast TGF-β response signature marked non-responders and TGF-β blockade restored intratumoral T-cell infiltration and immune checkpoint inhibitor (ICI) efficacy *in vivo* (Mariathasan et al., 2018). Consistently, single-cell studies have identified iCAFs associated with poor prognosis in MIBC, highlighting CAFs heterogeneity as a determinant of clinical outcome (Chen et al., 2020). Despite these advances, CAF-based prognostic models specifically tailored for BLCA progression remain limited. Most existing prognostic signatures focus on tumor cell-intrinsic features or immune infiltration patterns, overlooking the critical contribution of CAF heterogeneity to disease progression (Sjödahl et al., 2017; Robertson et al., 2017). Furthermore, the molecular mechanisms through which specific CAF-associated genes influence BLCA progression require further elucidation.

Ring finger protein 11 (RNF11) is a 154 amino-acid protein that was identified as a gene overexpressed in breast cancer (Burger et al., 1998; Seki et al., 1999). Subsequent studies have revealed that RNF11 is also overexpressed in tumors of the pancreas and colon (Subramaniam et al., 2003). RNF11 is a RING finger protein that is required for the A20 ubiquitin-editing complex to downregulate NF-κB signaling (Shembade and Harhaj, 2012; Shembade et al., 2009). Functionally, RNF11 facilitates A20-mediated deubiquitination to suppress TNF-induced NF-κB activation, with this regulatory mechanism being validated in neuronal and neuroblastoma cell lines (Shembade et al., 2009; Pranski et al., 2012). Additionally, RNF11 modulates epidermal growth factor receptor (EGFR), influencing cellular responses to growth signals (Santonico et al., 2014; Kostaras et al., 2012). While RNF11 dysregulation has been implicated in several cancers (Subramaniam et al., 2003; Mattioni et al., 2020), its specific role in BLCA progression and its relationship to CAF-mediated tumor progression remain unexplored.

In this study, based on the single-cell scRNA-seq dataset GSE267718 from GEO, we performed stringent quality control and unsupervised clustering to identify six major cell populations. By comparing MIBC with NMIBC, we delineated a CAF progression–associated gene set. We integrated this gene set with TCGA-BLCA bulk RNA-seq data and applied non-negative matrix factorization (NMF)-based consensus clustering in the training cohort to obtain 2 clusters (C1/C2) with significantly different survival. Using survival-associated candidate genes as input, we constructed a four-gene tumor-progression fibroblast cells riskscore (TPFR) prognostic model (*FOXA1, TBX3, LRIG1, RNF11*) via Least Absolute Shrinkage and Selection Operator (LASSO)–Cox and replicated its robust stratification and predictive performance in the external validation cohort E-MTAB-4321. Overall, higher TPFR scores predicted worse outcomes. Specifically, high RNF11 expression was associated with unfavorable prognosis. Knockdown of *RNF11* significantly suppressed proliferation, migration, and invasion of T24 and 5637 cells, supporting its pro-tumorigenic role. Our findings provide a clinically applicable prognostic tool and reveal RNF11 as a potential therapeutic target in BLCA.

## Results

### Single-cell clustering of BLCA and identification of progression associated CAF-signature genes

**FIGURE 1:**
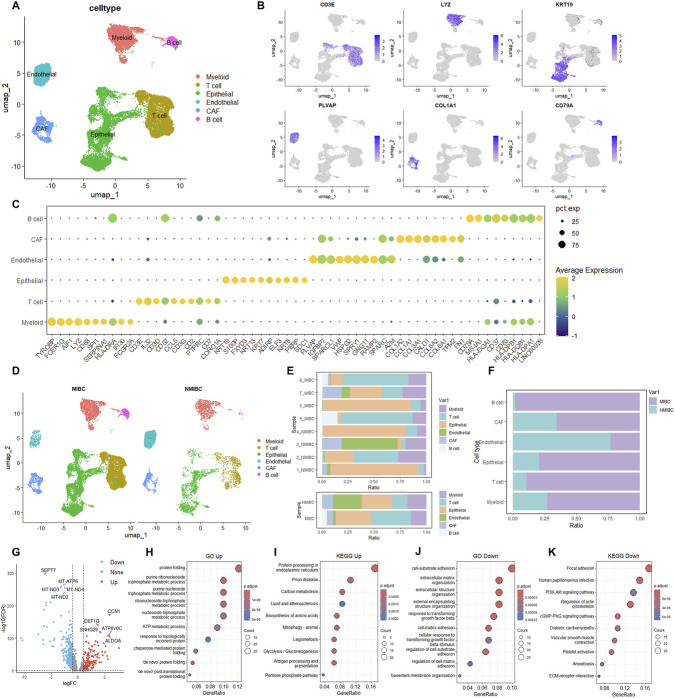
Single-cell analysis identifies CAF populations and progression-associated gene signatures in bladder cancer. **(A)** UMAP visualization of 30,428 cells from 8 BLCA patients (4 MIBCs, 4 NMIBCs) identifying six major cell populations. **(B)** Feature plots showing expression of cell type-specific markers. **(C)** Dot plot displaying expression patterns of canonical marker genes across all cell populations. Size indicates percentage of cells expressing the gene; color represents average expression level. **(D)** UMAP visualization stratified by MIBC and NMIBC samples. **(E)** Cell type distribution across individual patient samples. **(F)** Relative proportions of cell types in MIBC versus NMIBC samples. **(G)** Volcano plot of differentially expressed genes between MIBC and NMIBC CAFs (|log_2_FC| > 1, FDR <0.05), identifying 233 upregulated and 324 downregulated genes. **(H,I)** GO and KEGG pathway enrichment analysis of upregulated genes in MIBC CAFs. **(J,K)** GO and KEGG pathway enrichment analysis of downregulated genes in MIBC CAFs.

To delineate the cellular heterogeneity of the BLCA tumor microenvironment at single-cell resolution, we analyzed a published scRNA-seq data from GEO datasheets (GSE267718) comprising samples from 8 BLCA patients, including 4 MIBCs and 4 NMIBCs (Table 1). After quality control, 30,428 single cells were retained for subsequent analysis. Through unbiased clustering analysis, six distinct cell populations were identified, including myeloid cells (*LYZ*), T cells (*CD3E*), epithelial cells (*KRT19*), endothelial cells (*PLVAP*), CAFs (*COL1A1*), and B cells (*CD79A*) (Wang et al., 2021; Ma et al., 2022) (Figures 1A,B). The expression patterns of cell specific marker genes were visualized using a dot plot (Chen et al., 2020; Wang et al., 2021; Ma et al., 2022) (Figure 1C). Notably, myeloid cells exhibited high expressions of *TYROBP*, *FCER1G*, *LYZ*, and *CD68*, while *CD3E* and *IL32* were predominantly expressed in T cells. Fibroblast cells were characterized by elevated expression of *COL1A1*, *COL1A2*, and *TPM2*. Further analysis of BLCA tumor samples revealed that T cells, B cells, myeloid cells, epithelial cells, CAFs, and endothelial cells were all present in both MIBC and NMIBC (Figure 1D). Notably, CAFs were absent exclusively in samples 4, 5, and 6, while B cells showed a more restricted distribution, being undetected in samples 1, 2, 3, 6, and 7 (Figure 1E). Furthermore, B cells, CAFs, epithelial cells, T cells, and myeloid cells were predominantly derived from MIBC samples, whereas endothelial cells were mainly derived from NMIBC samples (Figure 1F).

**TABLE 1:** Demographic and clinical characteristics of BLCA scRNA-seq patient cohort.

Patient	Sex	Age	Surgical procedure	Disease grade	Disease stage	Treatment history
1	Male	90	TURBT	High-grade UC	NMI, papillary Ta	NA
2	Male	75	TURBT	High-grade UC	NMI, papillary T1	NA
3	Male	82	Cystectomy	High-grade UC with 20% squamous differentiation	MI, papillary T3a	NA
4	Male	82	Cystectomy	Small cell carcinoma (70%), squamous cell (20%), sarcomatoid carcinoma (9%), high-grade UC (1%)	MI, papillary T3b	BCG
5	Male	70	Cystectomy	High-grade UC with squamous differentiation	MI	NA
6	Male	64	TURBT	High-grade UC with squamous differentiation	MI	Pre-/post-chemotherapy treatment
7	Male	62	Cystectomy	High-grade UC	NMI, carcinoma *in situ*	NA
8	Female	63	TURBT	High-grade UC	NMI	NA

To explore the functional remodeling of CAFs during bladder cancer progression, we conducted a transcriptomic comparison of CAFs derived from MIBC and NMIBC samples. Differential expression analysis identified 557 differentially expressed genes (DEGs) (|log_2_FC| > 1, FDR <0.05), with 233 genes upregulated and 324 genes downregulated in MIBC-associated CAFs (Figure 1G; Supplementary Table S1). Gene Ontology (GO) functional enrichment analysis (Ashburner et al., 2000) revealed that these upregulated genes were significantly enriched in biological processes including protein folding, ATP metabolic process, and purine ribonucleoside/nucleoside triphosphate metabolic process. Kyoto Encyclopedia of Genes and Genomes (KEGG) pathway enrichment analysis (Kanehisa et al., 2023; Ogata et al., 1999) revealed that these upregulated genes were significantly enriched in protein processing in endoplasmic reticulum and glycolysis/gluconeogenesis (Figures 1H,I). In addition, GO analysis of the downregulated genes indicated an enrichment of cell-substrate adhesion, extracellular matrix/structure organization and cellular response to transforming growth factor beta stimulus. KEGG analysis revealed that the downregulated genes were mainly enriched in PI3K-AKT signaling pathway, cGMP-PKG signaling pathway, and ECM-receptor interaction (Figures 1J,K).

### Subtyping and prognostic evaluation of bladder cancer based on CAF-associated gene signatures

**FIGURE 2:**
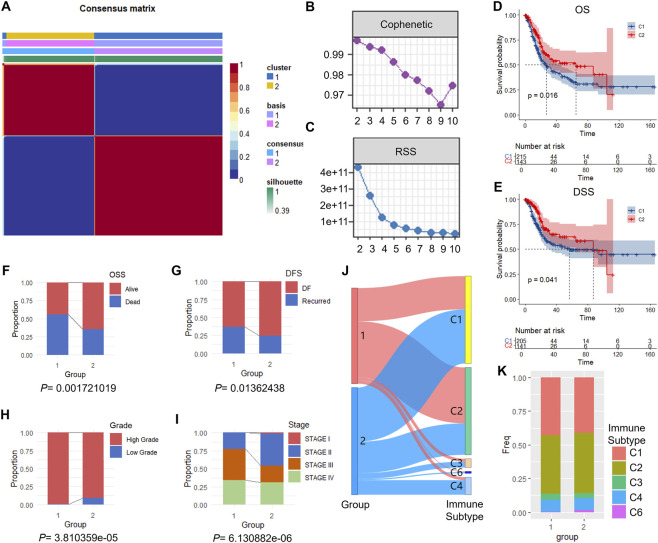
NMF clustering identifies CAF-associated molecular subtypes with distinct clinical outcomes. **(A)** Consensus matrix showing robust separation of TCGA-BLCA samples (n = 359) into 2 clusters based on 85 prognostic CAF-associated progression genes. **(B)** Cophenetic correlation coefficient plot showing optimal cluster number at k = 2. **(C)** Residual sum of squares (RSS) plot across different factorization ranks. **(D)** Kaplan-Meier curves showing overall survival (OS) differences between C1 and C2 clusters (*P* = 0.016, log-rank test). **(E)** DSS analysis between clusters (*P* = 0.041, log-rank test). **(F,G)** Distribution of overall survival status (OSS) and disease-free survival (DFS) between clusters (*P* = 0.001 and *P* = 0.013, respectively; chi-square test). **(H,I)** Comparison of histologic grade and clinical stage distribution between clusters (*P* = 3.8 × 10^−5^ and *P* = 6.1 × 10^−5^, respectively; chi-square test). **(J)** Sankey diagram showing immune subtype transitions between clusters. **(K)** Relative proportions of immune subtypes in C1 versus C2.

Bulk RNA-seq data of 359 BLCA samples were obtained from TCGA datasheet. The expression profiles of 557 CAF-associated progression genes were used for further analysis. Univariate Cox regression analysis identified 85 genes significantly associated with prognosis (*P* < 0.05). Using NMF clustering, the samples were clustered into two molecular subtypes—Cluster 1 (C1) and Cluster 2 (C2) (Figures 2A–C). Survival analyses revealed that C2 exhibited better overall survival (OS) and disease-specific survival (DSS) compared to C1 (Figures 2D,E). Consistently, clinical data showed more favorable overall survival status (OSS) and disease-free survival (DFS) in C2. C2 also had lower histologic grade and earlier clinical stage, which was consistent with its superior prognosis (Figures 2F–I). Thorsson et al. defined six immune subtypes based on immune-related gene expression features (Thorsson et al., 2018), named type 1 (Wound Healing), type 2 (IFN-γ Dominant), type 3 (Inflammatory), type 4 (Lymphocyte Depleted), type 5 (Immunologically Quiet), and type 6 (TGF-β Dominant). We compared the immune subtype distributions between C1 and C2. Both subtypes were composed of type 1, 2, 3, 4 and 6 phenotypes. Specifically, the proportion of type 6 was higher in C2 (Figures 2J,K).

### C1 shows higher stromal, immune, and ESTIMATE scores than C2

**FIGURE 3:**
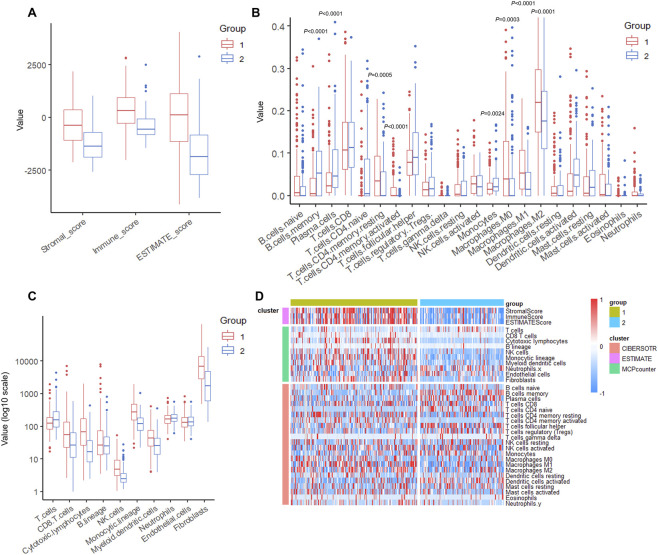
C1 cluster exhibits elevated stromal and immune infiltration signatures. **(A)** Comparison of StromalScore, ImmuneScore, and ESTIMATEScore between C1 and C2 clusters. *P* < 0.001 for all comparisons (Wilcoxon rank-sum test). **(B)** CIBERSORT analysis of 22 immune cell populations showing differential infiltration patterns between clusters. *P* values indicated for significantly different cell types (Wilcoxon rank-sum test). **(C)** MCPcounter scores for 10 immune and stromal cell populations. *P* < 0.001 for CD8^+^ T cells, cytotoxic lymphocytes, NK cells, and monocytic lineages (Wilcoxon rank-sum test). **(D)** Integrated heatmap visualization of immune infiltration profiles from CIBERSORT, ESTIMATE, and MCPcounter algorithms across C1 and C2 clusters.

We calculated the StromalScore, ImmuneScore, and ESTIMATEScore (Yoshihara et al., 2013) of the 2 clusters. These three scores were significantly higher in C1 compared to C2 (Figure 3A). Further CIBERSORT deconvolution (Newman et al., 2015) of specific immune cell populations revealed distinct infiltration patterns between the 2 clusters. Among the 22 immune cell types analyzed, we observed that C1 exhibited significantly higher infiltration scores among multiple cell types including CD4^+^ memory activated T cells, M0 M1 M2 macrophages, whereas scores of memory B cells, plasma cells, and monocytes were lower in C1 (Figure 3B, *P* < 0.05 for all comparisons). Moreover, we employed MCPcounter scoring (Becht et al., 2016) to characterize the immune infiltration landscape. Among the 10 cell populations evaluated, C1 cluster exhibited significantly higher scores of immune cells including CD8^+^ T cells, cytotoxic lymphocytes, NK cells, and monocytic lineages, compared to C2 (Figure 3C, *P* < 0.001 for all comparisons). Finally, we integrated the results from all three algorithms and visualized them using a composite heatmap, providing a comprehensive overview of the distinct immune landscape characterizing the clusters (Figure 3D).

### Chemotaxis-related pathways are enriched in C1

**FIGURE 4:**
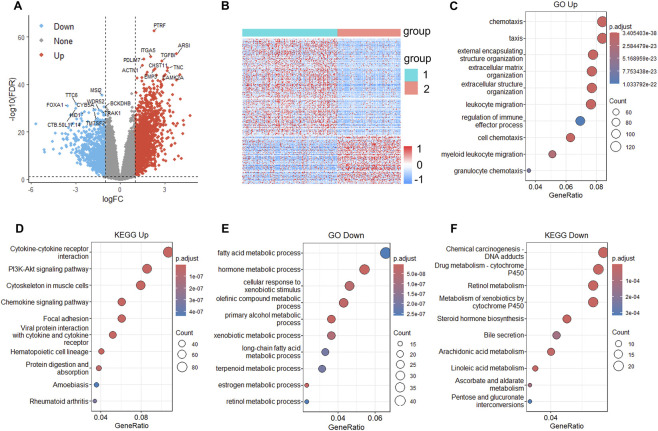
Differential expression analysis reveals immune-related and metabolic pathway enrichment between clusters. **(A)** Volcano plot showing 1,681 upregulated and 765 downregulated genes in C1 versus C2 (|log_2_FC| > 1, FDR <0.05). **(B)** Heatmap of 2,446 DEGs showing distinct expression patterns between clusters. **(C,D)** GO and KEGG enrichment analysis of upregulated genes in C1. **(E,F)** GO and KEGG analysis of downregulated genes in C1. Gene ratio and adjusted *P* values are indicated.

To delineate the biological characteristics between C1 and C2, we performed differential expression analysis using limma (Ritchie et al., 2015). We identified 1,681 significantly upregulated and 765 downregulated genes in C1 relative to C2 (|log_2_FC| > 1; FDR <0.05). The volcano plot highlights several notably dysregulated genes, including *PTRF*, *TGFBI*, and *ARSI* among the upregulated genes, and *FOXA1*, *WDR52*, and *MSI2* among the downregulated genes in C1 (Figure 4A). The heatmap visualization of these 2,446 DEGs clearly shows distinct expression patterns between the 2 clusters (Figure 4B). GO and KEGG analysis of upregulated genes in C1 revealed significant enrichment in pathways associated with cell chemotaxis, leukocyte migration, extracellular matrix and structure organization, PI3K-Akt signaling pathway (Figures 4C,D). The enrichment of immune-related pathways in C1 consistent with its higher ImmuneScore, StromalScore, and ESTIMATEScore. For downregulated genes in C1, GO analysis identified enrichment in fatty acid metabolic process, hormone metabolic process, and various other metabolic processes (Figure 4E). KEGG analysis revealed enrichment in chemical carcinogenesis-DNA adducts, retinol metabolism, and steroid hormone biosynthesis pathways (Figure 4F).

### Construction and validation of the TPFR prognostic risk model

**FIGURE 5:**
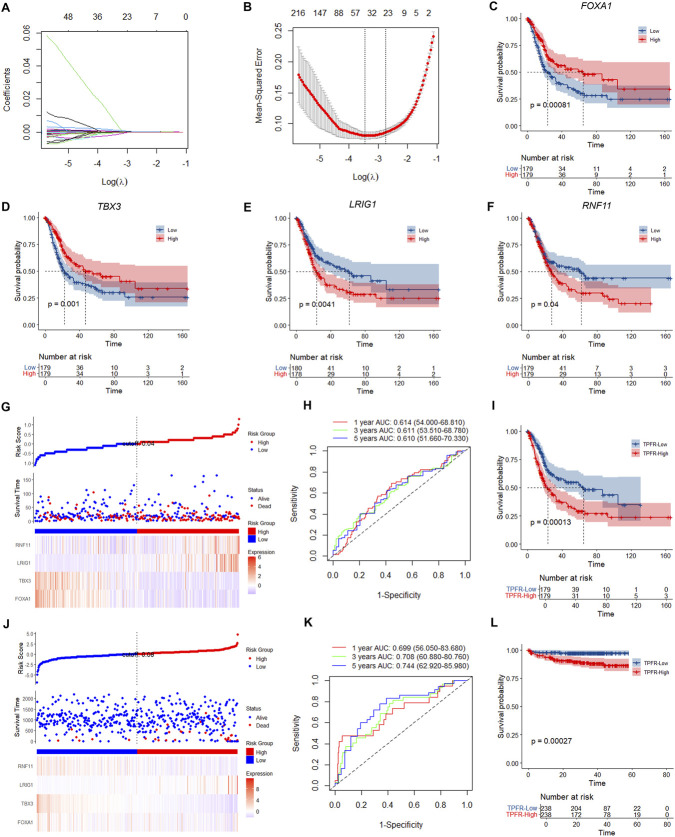
Construction and validation of the four-gene TPFR prognostic model. **(A)** LASSO coefficient profiles of 569 prognostic genes. **(B)** Cross-validation for optimal lambda selection (λ = 0.03183554) with mean-squared error. **(C–F)** Kaplan-Meier survival curves for individual model genes: *FOXA1* (*P* = 0.00081), *TBX3* (*P* = 0.001), *LRIG1* (*P* = 0.0041), and *RNF11* (*P* = 0.04). **(G)** Risk score distribution, survival status, and gene expression heatmap in TCGA training cohort (n = 359). **(H)** Time-dependent ROC curves showing AUCs of 0.614, 0.611, and 0.610 at 1-, 3-, and 5-year timepoints in training cohort. **(I)** Kaplan-Meier analysis showing significantly worse OS in high-risk group (*P* = 0.00013, log-rank test). **(J–L)** Validation in E-MTAB-4321 cohort (n = 476) showing consistent risk stratification, improved predictive performance (AUCs: 0.699, 0.708, 0.744), and significant survival difference (*P* = 0.00027, log-rank test).

To construct a tumor progressing-related prognostic model, we first performed univariable Cox regression and identified 569 prognosis-associated genes at *P* < 0.05 (Supplementary Table S2), which were subjected to Lasso-Cox regression. As λ increased, gene coefficients shrank toward zero; cross-validation across λ values identified λ = 0.03183554 as optimal, yielding a 47-gene model for subsequent Cox modeling (Figures 5A,B). To prioritize features with clinical value, we applied akaike information criterion (AIC)-based selection and obtained four genes: *FOXA1*, *TBX3*, *LRIG1*, and *RNF11*. *RNF11* and *LRIG1* were negatively associated with prognosis (higher expression correlated with worse survival), while *FOXA1* and *TBX3* were positively associated with prognosis (Figures 5C–F).

Based on the four-gene signature (*FOXA1*, *TBX3*, *LRIG1*, *RNF11*), we constructed the TPFR prognostic model. The TPFR score for each patient was calculated using the Cox regression coefficients from the training cohort. In the TCGA-BLCA training cohort (n = 359), patients were stratified into high-risk and low-risk groups based on the median risk score. The risk score distribution, survival status, and expression heatmap clearly revealed distinct patterns between the two groups (Figure 5G). Time-dependent Receiver Operating Characteristic (ROC) analysis showed robust predictive performance with areas under the curve (AUC) of 0.614 at 1 year, 0.611 at 3 years, and 0.610 at 5 years (Figure 5H). Kaplan-Meier survival analysis revealed significantly poorer OS in the high-risk group compared to the low-risk group (Figure 5I). The model’s performance was validated in the independent E-MTAB-4321 cohort (n = 476). Consistent with the training cohort, the risk score distribution and gene expression patterns showed clear stratification (Figure 5J). The model maintained predictive accuracy with AUCs of 0.699, 0.708, and 0.744 at 1, 3, and 5 years, respectively (Figure 5K). Consistently, the high-risk group displayed significantly worse survival outcomes (Figure 5L). Overall, these results show that the TPFR model provides robust and reproducible prognostic prediction for BLCA patients across independent cohorts.

### High TPFR score is associated with tumor-related pathways

**FIGURE 6:**
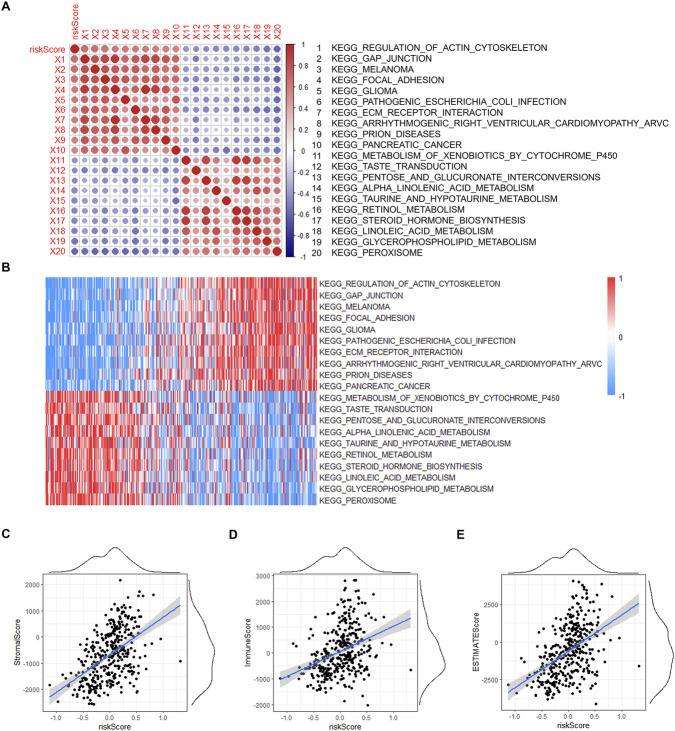
TPFR score correlates with tumor-related pathways and microenvironmental features. **(A)** Correlation matrix between TPFR score and KEGG pathway enrichment scores across TCGA-BLCA samples. **(B)** Heatmap visualization of pathway activity scores stratified by risk groups. **(C–E)** Linear regression analysis showing positive correlations between TPFR score and StromalScore (R = 0.50, *P* < 0.001), ImmuneScore (R = 0.36, *P* < 0.001), and ESTIMATEScore (R = 0.47, *P* < 0.001). All correlations assessed by Pearson correlation coefficient.

We performed single-sample gene set enrichment analysis (ssGSEA) using the GSVA R package (Hänzelmann et al., 2013) to calculate pathway activity scores for each patient. We systematically evaluated the correlation between the TPFR and enrichment scores of KEGG pathways across the TCGA-BLCA cohort, which showed significant associations between the TPFR score and multiple cancer-related pathways (Figure 6A). Pathway enrichment analysis revealed distinct patterns of correlation with the TPFR score. Pathways involved in cellular structure and communication, including regulation of actin cytoskeleton, focal adhesion, ECM-receptor interaction, and gap junction, showed positive correlations with the TPFR score. These pathways regulate diverse cellular functions ranging from cell migration to tissue architecture maintenance. Additionally, several tumor type-specific pathways (melanoma, glioma, and pancreatic cancer) also showed positive correlations with higher risk scores. Metabolic pathways, particularly those involved in xenobiotic metabolism and lipid metabolism, were negatively correlated with the TPFR score (Figures 6A,B). We then computed StromalScore, ImmuneScore, and ESTIMATEScore, all of which were positively correlated with the TPFR score based on linear regression and Pearson correlation analyses (Figures 6C–E).

### TPFR model shows superior predictive performance in >60 BLCA patients

**FIGURE 7:**
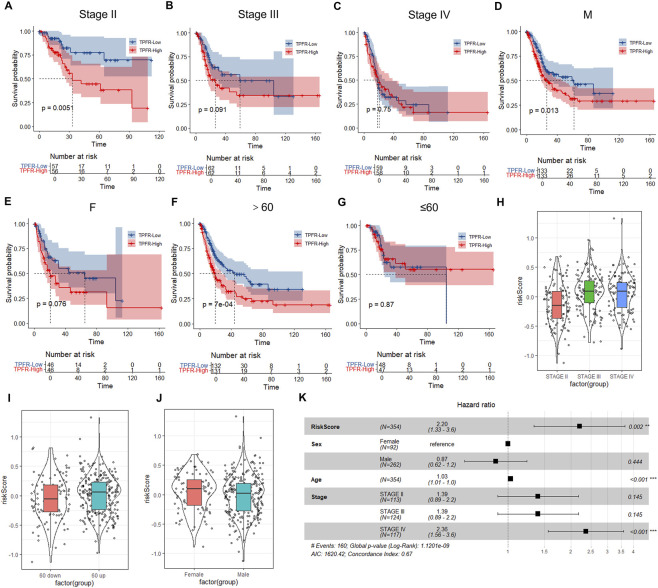
Stratified analysis reveals superior TPFR model performance in specific patient subgroups. **(A–C)** Kaplan-Meier curves showing prognostic value across tumor stages, with significant stratification in stage II (*P* = 0.0051) but not in advanced stages. **(D,E)** Sex-stratified analysis demonstrating significant prognostic value in males (*P* = 0.013) but not females (*P* = 0.076). **(F,G)** Age-stratified analysis showing superior performance in patients >60 years (*P* = 7 × 10^−4^) versus ≤60 years (*P* = 0.87). **(H–J)** Distribution of TPFR scores across clinical subgroups. *P* values from Wilcoxon rank-sum test. **(K)** Forest plot of multivariate Cox regression identifying TPFR score (HR = 2.20, *P* = 0.002), age (HR = 1.03, *P* < 0.001), and high stage (HR = 2.36, *P* < 0.001) as independent prognostic factors.

To evaluate the generalizability of the TPFR model across diverse patient subgroups stratified by tumor stage, sex, and age, we further validated the model in the indicated groups. The results showed that the TPFR score was negatively correlated with prognosis in patients with low-stage BLCA, while no significant correlation upon tumor stage increased (Figures 7A–C). Moreover, higher TPFR scores were negatively correlated with prognosis in male patients, whereas no significant correlation with prognosis was observed in female patients (Figures 7D,E). In patients >60 years old, TPFR scores showed a significant negative correlation with prognosis, while no significant prognostic correlation was observed in patients ≤60 years old (Figures 7F,G). These results suggest that in clinical application, the TPFR model is more suitable for low-stage, elderly (>60 years), and male BLCA patients. Analysis of the TPFR scores in different tumor stage showed an increase with tumor stage progression (Figure 7H). Patients >60 years old had higher TPFR scores compared to those ≤60 years old (Figure 7I), which is consistent with the clinical observation of poorer prognosis in elderly BLCA patients. Between sexes, females exhibited higher TPFR scores, consistent with the clinically observed poorer prognosis in female BLCA patients (van der Heijden et al., 2025) (Figure 7J). In multivariate Cox regression analysis, TPFR score, age, and high stage were identified as independent risk factors for BLCA (Figure 7K).

### RNF11 promotes proliferation, migration and invasion of BLCA

**FIGURE 8:**
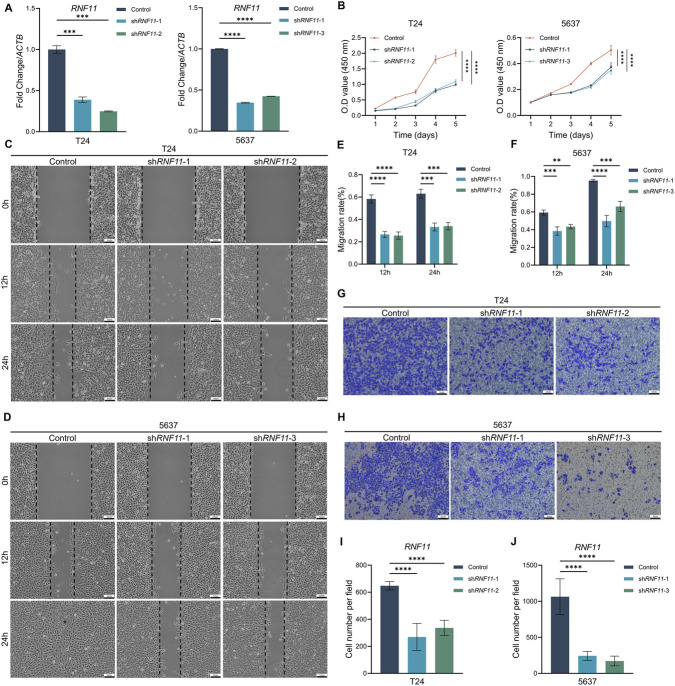
RNF11 promotes bladder cancer cell proliferation, migration, and invasion. **(A)** qPCR validation of *RNF11* knockdown efficiency in T24 and 5637 cells. **(B)** CCK-8 proliferation assays revealing significant growth inhibition following *RNF11* knockdown over 5 days. **(C,D)** Representative wound healing images at 0, 12, and 24 h post-scratch. Scale bars, 1 mm. **(E,F)** Quantification of migration rates showing ∼50% reduction at 24 h in both cell lines. **(G,H)** Representative transwell invasion assay images. Scale bars, 1 mm. **(I,J)** Quantification of invaded cells showing 60%–70% reduction following *RNF11* knockdown. Data shown as means ± SEM from three independent experiments.

To functionally validate the findings above, *RNF11* was knocked down via shRNA-mediated lentiviral infection (sh*RNF11*) in T24 and 5637 cells. qPCR analysis confirmed successful knockdown efficiency (Figure 8A). Cell proliferation assays using CCK-8 showed that *RNF11* knockdown significantly suppressed cell growth in both cell lines. In T24 cells, both sh*RNF11*-1 and sh*RNF11*-2 showed reduced proliferation during the 5-day culturing. Similarly, *RNF11* knockdown significantly suppressed growth of 5637 cells (Figure 8B). Wound healing assays revealed that *RNF11* knockdown markedly impaired cell migration capacity. In T24 cells, the migration rate at 12 h was reduced from 55% in control cells to approximately 25% in both sh*RNF11*-1 and sh*RNF11*-2 groups. At 24 h, control cells achieved nearly 70% wound closure, while *RNF11*-knockdown cells showed only 35%–40% closure (Figures 8C,E). Similar inhibitory effects were observed in 5637 cells, where *RNF11* knockdown reduced migration rates by approximately 40% at 12 h and 50% at 24 h compared to control (Figures 8D,F). Transwell invasion assays further confirmed the role of *RNF11* in promoting invasive capacity. In T24 cells, *RNF11* knockdown reduced the number of invading cells from approximately 650 cells per field in the control group to 250–300 cells per field in sh*RNF11* groups (Figures 8G,I). In 5637 cells, invasion was similarly suppressed, with invaded cell numbers decreasing from approximately 1,050 in control to 300–250 in *RNF11*-depleted groups (Figures 8H,J). Collectively, these functional studies showed that *RNF11* plays a critical role in promoting bladder cancer cell proliferation, migration, and invasion, supporting its potential as a therapeutic target in BLCA.

### RNF11 knockdown reprograms developmental and inflammatory pathways in BLCA cells

**FIGURE 9:**
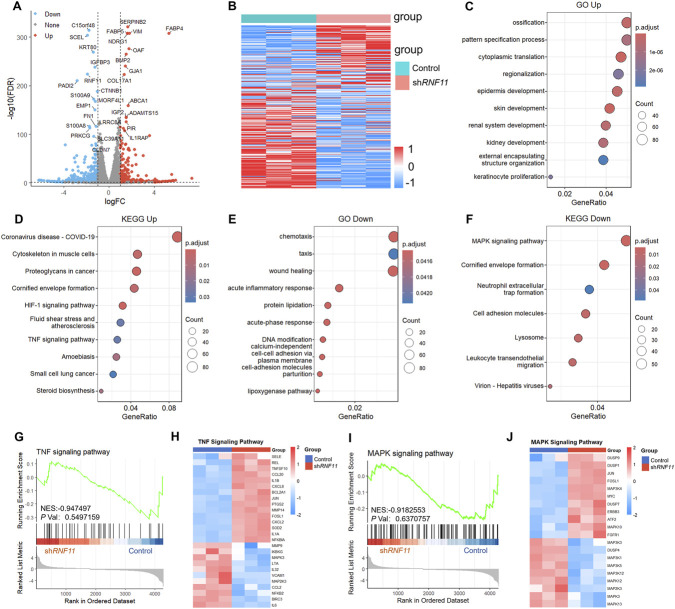
Transcriptomic profiling reveals RNF11-mediated regulation of developmental and inflammatory programs. **(A)** Volcano plot showing 1,820 upregulated and 2,483 downregulated genes following *RNF11* knockdown in 5637 cells (|log_2_FC| > 0.6, FDR <0.05). **(B)** Heatmap of DEGs between control and sh*RNF11* samples (n = 3 per group). **(C,D)** GO and KEGG enrichment of upregulated genes. **(E,F)** GO and KEGG analysis of downregulated genes. **(G)** GSEA plot showing negative enrichment of TNF signaling pathway (NES = −0.947497, *P* = 0.5497159). **(H)** GSEA analysis of TNF signaling pathway with detailed gene expression patterns across control and sh*RNF11* groups. **(I)** GSEA plot showing negative enrichment of MAPK signaling pathway (NES = −0.9182553, *P* = 0.6370757). **(J)** GSEA analysis of MAPK signaling pathway with detailed gene expression patterns across control and sh*RNF11* groups.

To elucidate how RNF11 regulated proliferation of bladder cancer cells, we performed bulk RNA sequencing on control and sh*RNF11* 5637 cells. Differential expression analysis revealed substantial transcriptional alterations following *RNF11* knockdown. Using the criteria of |log_2_FC| > 0.6 and FDR <0.05, we identified 1,820 significantly upregulated genes and 2,483 downregulated genes. The volcano plot visualization revealed several prominently altered genes following *RNF11* knockdown. Among the most significantly upregulated genes were *FABP4, SERPINB2, VIM, NDRG1, OAF, BMP2, GJA1,* and *COL17A1*. In addition, the most significantly downregulated genes included *RNF11, C15orf48, SCEL, KRT80, IGFBP3, PADI2, S100A9,* and *EMP1* (Figure 9A). Heatmap analysis of DEGs revealed distinct expression patterns between control and sh*RNF11* samples, confirming the transcriptomic alterations induced by *RNF11* (Figure 9B). GO enrichment analysis of upregulated genes revealed significant enrichment in developmental and morphogenic processes. The most significantly enriched biological processes included ossification, pattern specification process, cytoplasmic translation, regionalization, epidermal development, skin development, renal system development, and kidney development (Figure 9C). KEGG pathway analysis of upregulated genes identified enrichment in multiple disease and signaling pathways, including cytoskeleton in muscle cells, proteoglycans in cancer, cornified envelope formation, HIF-1 signaling pathway, and TNF signaling pathway (Figure 9D). For downregulated genes, GO enrichment analysis revealed significant alterations in cellular migration and inflammatory responses. The top enriched biological processes included chemotaxis, taxis, wound healing, and acute inflammatory response (Figure 9E). KEGG pathway analysis of downregulated genes showed enrichment in MAPK signaling pathway, cornified envelope formation, neutrophil extracellular trap formation, cell adhesion molecules, lysosome, and leukocyte transendothelial migration (Figure 9F).

Gene Set Enrichment Analysis (GSEA) was performed to examine the enrichment of specific KEGG pathways in *RNF11* knockdown cells (Subramanian et al., 2005). The TNF signaling pathway showed negative enrichment (NES = −0.947497, *P* = 0.5497159), with the enrichment plot displaying a downward trend in the running enrichment score (Figure 9G). The accompanying heatmap revealed complex transcriptional alterations within the TNF signaling pathway. *REL*, *IL1B*, *NFKBIA*, and *BCL2A1* showed increased expression in sh*RNF11* samples, while *VCAM1*, *NFKB2*, *MAPK3*, *BIRC3,* and *IL6* displayed reduced expression compared to controls (Figure 9H). Similarly, the MAPK signaling pathway exhibited negative enrichment (NES = −0.9182553, *P* = 0.6370757) following *RNF11* knockdown (Figure 9I). The expression heatmap revealed differential regulation of MAPK pathway components, with upregulation of *MAP3K4*, *JUN*, *MYC*, and *MAPK10* in sh*RNF11* cells, whereas *MAP3K3*, *DUSP4*, and several MAPK family members showed decreased expression (Figure 9J).

## Discussion

This study provides a comprehensive framework for bladder cancer risk stratification based on CAF-associated transcriptional signatures, addressing a critical gap in current prognostic approaches. By integrating single-cell transcriptomics with bulk RNA sequencing data, we developed the TPFR model that demonstrates robust prognostic performance across independent cohorts. Our functional validation of *RNF11* provides mechanistic insights into bladder cancer progression.

Single-cell analysis revealed distinct transcriptional programs in CAFs from MIBC versus NMIBC patients, with MIBC-associated CAFs enriched for protein folding, ATP metabolism, and glycolysis/gluconeogenesis, consistent with metabolic reprogramming described for activated CAFs (Kalluri, 2016). We also observed alterations in genes related to ECM organization and TGF-β responses in MIBC-associated CAFs. This is consistent with a higher representation of iCAFs (low α-SMA, inflammatory secretome) described in pancreatic cancer (Öhlund et al., 2017). We therefore interpret these data as suggestive of a shift toward more metabolically active and immunomodulatory CAF phenotypes as disease progresses to MIBC, while acknowledging that spatially resolved and functional assays will be required to confirm this in bladder cancer. The NMF-based clustering identified two distinct CAF-associated molecular clusters with significant survival differences. Notably, the C1, associated with poorer prognosis, showed higher stromal and immune scores, as well as enrichment for immunosuppressive pathways. Given the established pro-tumorigenic role of stromal cells, the enrichment of stromal in C1 was consistent with the poorer prognosis of C1. Nevertheless, the higher immune score and poorer prognosis in C1 contrast with the anticipated anti-tumor effects of immune response. This observation may be contributed by the concept of “immune-excluded” phenotypes in bladder cancer, where abundant immune infiltration fails to translate into effective anti-tumor immunity due to CAF-mediated barriers (Mariathasan et al., 2018; Wang et al., 2018). Furthermore, the immune system constitutes a complex and integrated entity, comprising both anti-tumor components such as effector CD8^+^ T cells and pro-tumor elements including regulatory T cells (Tregs) and M2 macrophages (Fridman et al., 2017; Mantovani et al., 2017; Tanaka et al., 2016). The elevated immune score observed in subgroup C1 may be attributable to the predominance of immunosuppressive cell populations, which is consistent with the subsequent observation of higher M2 macrophage infiltration scores, and enrichment of immunosuppressive pathways.

The TPFR model showed consistent prognostic performance across training and validation cohorts, with particularly strong discrimination in elderly patients (>60 years) and early-stage disease. The superior performance in early-stage disease suggests potential utility for identifying patients at high risk of progression who might benefit from more aggressive surveillance or adjuvant therapy (Sylvester et al., 2021).

Among the 4 TPFR genes, *RNF11* merits prioritization on empirical and mechanistic grounds. It was nominated from the scRNA-seq progression gene in BLCA and associated with adverse outcome in the training cohort. As an E3 ubiquitin ligase, RNF11 provides tractable points of intervention at the level of enzyme activity or substrate engagement, whereas the other panel members are transcriptional or negative-regulatory factors with limited druggability in current clinical frameworks. Independent reports of *RNF11* dysregulation across multiple malignancies further support its disease relevance and translational potential (Kitching et al., 2003; Rao et al., 2020; Burger et al., 2006). Our functional data support a pro-tumorigenic role for *RNF11* in BLCA, as its knockdown decreased cellular proliferation, migration, and invasion of tumor cells. Transcriptomic profiling after *RNF11* knockdown revealed alterations of developmental and differentiation programs in BLCA cells.

Notably, while *RNF11* expression positively correlated with risk scores in the training cohort, we observed an inverse relationship in the validation cohort. Several explanations may account for this discrepancy. Cohort composition likely contributes, since E-MTAB-4321 contains a larger fraction of NMIBC (Ta/Tis/T1) than TCGA-BLCA (≥T2), these distinct stage compositions could lead to different expression patterns of *RNF11*. In addition, as an E3 ubiquitin ligase, RNF11 operates primarily through post-translational modification (PTM) (Connor and Seth, 2004), mRNA expression may incompletely capture its functional state. This apparent paradox may reflect different regulatory mechanisms or post-translational modifications affecting RNF11 function in different patient populations, warranting further investigation into RNF11 regulation and activity rather than expression alone (Chen and Matesic, 2007).

The TPFR model showed moderate predictive accuracy, with AUC values ranging from 0.610–0.614 in the TCGA-BLCA training cohort and 0.699–0.744 in the E-MTAB-4321 validation cohort. While these values indicate modest discriminative ability, it is noteworthy that the model showed consistent prognostic stratification across both independent cohorts, with statistically significant separation of high- and low-risk groups in Kaplan-Meier analyses. The improved performance in the validation cohort (AUC up to 0.744 at 5 years) suggests that the model may perform differentially depending on cohort composition and disease stage distribution.

## Methods

### Single-cell RNA sequencing data acquisition and preprocessing

We obtained the scRNA-seq data from GEO datasheets (GSE267718) involving eight patients (4 NMIBC tumors, 4 MIBC tumors).

Raw sequencing data were processed and quality-controlled using the Seurat (v5.3.0) R package. Rigorous filtering criteria were applied to retain high-quality cells for downstream analyses. Specifically, cells were excluded if they expressed fewer than 200 or more than 5,000 unique genes, had fewer than 1,000 or more than 25,000 unique molecular identifiers (UMIs), or exhibited a mitochondrial gene content exceeding 15%, as high mitochondrial content may indicate low-quality or dying cells. In addition, genes detected in fewer than three cells were removed. After quality control, a total of 30,428 high-quality single cells were retained, including 23,274 cells from MIBC patients and 7,154 cells from NMIBC patients.

### Cell clustering and annotation

Following normalization of gene expression matrices using the “LogNormalize” method, highly variable genes were identified for dimensionality reduction via principal component analysis (PCA). The top 20 principal components were selected for subsequent unsupervised clustering. Nonlinear dimensionality reduction was performed using uniform manifold approximation and projection (UMAP) to visualize the transcriptional landscape of all high-quality single cells. Cell-type annotation was conducted by examining the expression of canonical marker genes that define major cell lineages.

### Differential expression analysis

Differential expression analysis was performed at both single-cell and bulk transcriptome levels. For single-cell CAF-related populations, differential expression between MIBC and NMIBC samples was identified using the FindMarkers function in Seurat (v5.3.0) with Wilcoxon rank-sum test. All genes meeting the criteria of |log_2_FC| > 1 and FDR <0.05 were considered differentially expressed genes (DEGs). Among the 1,623 CAF-associated genes identified through scRNA-seq data, we identified 557 genes that were differentially expressed between MIBC and NMIBC. For bulk RNA-seq analysis of clusters (C1 vs. C2) identified by NMF clustering, differential expression was performed on TCGA data (n = 359) using the Limma package (v3.64.1) (Ritchie et al., 2015). RNA-seq count data were transformed using the voom function to convert counts to log2-counts per million (log2-CPM) with associated precision weights, accounting for the mean-variance relationship in RNA-seq data. Linear models were then fitted using the lmFit function, contrasts between C1 and C2 were defined using makeContrasts and applied with contrasts.fit, and empirical Bayes moderation was applied with the eBayes function to stabilize variance estimates across genes. Genes with |log_2_FC| > 1 and FDR <0.05 were considered significantly differentially expressed between the 2 clusters. For *RNF11* knockdown in 5637 bladder cancer cells, RNA sequencing and differential expression analysis were performed using standard RNA-seq analysis pipelines. Genes with |log_2_FC| > 0.6 and FDR< 0.05 were considered differentially expressed. All volcano plots were generated using ggplot2 (v3.5.2) for gene label positioning to prevent overlapping.

### Pathway enrichment analysis

Gene Ontology (GO) (Ashburner et al., 2000) and Kyoto Encyclopedia of Genes and Genomes (KEGG) (Ogata et al., 1999) pathway enrichment analyses were conducted using the clusterProfiler package (v4.16.0). Gene symbols were first converted to ENTREZ IDs using the bitr function with the org.Hs.eg.db annotation database (v3.21.0). Enriched pathways were ranked by gene ratio and adjusted *P* values, with pathways meeting FDR <0.05 considered significantly enriched. Gene Set Enrichment Analysis (GSEA) (Subramanian et al., 2005) was performed using the gseKEGG function in clusterProfiler. Normalized enrichment scores (NES) and FDR q-values were calculated for each pathway. Single-sample Gene Set Enrichment Analysis (ssGSEA) was conducted using the GSVA package (v2.2.0) to calculate pathway activity scores for individual samples.

### Kaplan-Meier survival analysis

Kaplan-Meier survival analysis was performed using the R packages survival (v3.8.3) and survminer (v0.5.0). For the CAF identified by NMF clustering, patients were stratified into C1 and C2 groups based on their cluster assignment. For individual CAF-related genes and the TPFR risk score model, patients were divided into high and low expression groups using the median expression value as the cutoff threshold. Overall survival (OS) and disease-specific survival (DSS) were analyzed as primary endpoints. Survival curves were estimated using the Kaplan-Meier method, and differences between groups were assessed using the log-rank test. Univariate Cox proportional hazards regression analysis was performed to calculate hazard ratios (HR) with 95% confidence intervals (CI) for each gene and risk group.

For the prognostic gene signature screening, univariate Cox regression was applied to all 557 CAF-related genes in the training cohort (n = 359), and genes with *P* < 0.05 were retained for subsequent LASSO-Cox regression analysis. The TPFR risk score was calculated as a linear combination of the expression levels of the four signature genes weighted by their respective Cox regression coefficients. Patients in both training (TCGA-BLCA, n = 359) and validation (E-MTAB-4321, n = 476) cohorts were stratified into TPFR-high and TPFR-low groups based on the median risk score. Survival curves were visualized using the ggsurvplot function with 95% confidence intervals, median survival lines, and risk tables. All statistical tests were two-sided, and *P* < 0.05 was considered statistically significant.

### Bulk RNA-seq data acquisition from TCGA-BLCA and E-MTAB-4321-ArrayExpress

Transcriptome data from The Cancer Genome Atlas (TCGA) BLCA datasets were obtained from UCSC XENA platform (https://xena.ucsc.edu/). Clinical and survival annotations were taken from the TCGA Pan-Cancer Atlas resource. The E-MTAB-4321 dataset was downloaded from ArrayExpress database (https://www.ebi.ac.uk/arrayexpress/).

Data processing was performed using R (v4.5.0). The preprocessing workflow included three steps: (Sung et al., 2021): Clinical data filtering: Samples lacking essential clinical information (overall survival time and vital status) were excluded from downstream analysis. (Bray et al., 2024). Gene identifier standardization: Gene identifiers were converted to official gene symbols using the org.Hs.eg.db package (v3.21.0) and the bitr function from clusterProfiler package (v4.16.0). For TCGA data, Ensembl gene IDs were mapped to HUGO gene symbols; for E-MTAB-4321, probe IDs were converted to gene symbols according to platform annotation files. (Lopez-Beltran et al., 2024). Duplicate gene resolution: When multiple transcripts or probes mapped to the same gene symbol, the entry with the highest mean expression across all samples was retained to ensure representative gene-level quantification. After preprocessing, 359 BLCA samples from TCGA and 476 samples from E-MTAB-4321 were included in subsequent analyses.

### Consistent cluster analysis of CAF-associated genes in TCGA

From the 557 CAF-associated genes identified through integration of single-cell analysis and published CAF signatures, univariate Cox regression analysis was performed to identify prognostic genes in the TCGA-BLCA cohort. Genes with *P* < 0.05 were considered prognostically significant, yielding 85 genes for subsequent analysis. NMF consensus clustering was applied to classify the 359 TCGA-BLCA samples into molecular clusters based on the expression profiles of these 85 prognostic CAF-associated genes. The analysis was performed using the NMF package (v0.28) in R. To determine the optimal number of clusters, we evaluated factorization ranks from 2 to 10 with 50 iterations per rank. Multiple metrics were assessed including cophenetic correlation coefficient, dispersion, explained variance ratio, residuals, residual sum of squares (RSS), silhouette width, and sparseness. The optimal cluster number was determined when the cophenetic correlation coefficient began to decline (from 0.993 at k = 2 to lower values), the rate of RSS decrease plateaued, and the consensus matrix showed clear block-diagonal structure. Based on these comprehensive metrics, k = 2 was selected as the optimal cluster number, demonstrating high consensus stability (cophenetic correlation = 0.993) and clear separation between clusters as visualized in the consensus heatmap. The resulting 2 CAF-associated clusters (C1 and C2) showed distinct molecular profiles. Kaplan-Meier survival analysis with log-rank test was performed to compare OS and DSS between clusters.

### Lasso cox regression analysis

To construct a robust prognostic signature, we employed the LASSO Cox regression analysis. Initially, DEGs between CAF-associated clusters with statistical significance (*P* < 0.05) and biological relevance (|log_2_FC| > 1) were identified using the Limma package in R. Genes significantly associated with overall survival (*P* < 0.05) in univariate Cox proportional hazards analysis were selected as candidate prognostic genes for subsequent LASSO modeling. The 359 TCGA-BLCA samples were used as the training cohort.

LASSO Cox regression was performed using the glmnet package (v4.1.9) in R with 10-fold cross-validation to determine the optimal penalty parameter (λ). The optimal λ value was selected using the “lambda.min” criterion, which corresponds to the value that gives minimum mean cross-validated error. To ensure model stability and reproducibility, the cross-validation procedure was repeated with different random seeds, and the most frequently selected features were retained. Subsequently, backward stepwise regression using the AIC was applied to further refine the gene signature using the MASS package, ensuring the most parsimonious model with optimal predictive performance. The final prognostic signature was constructed based on the linear combination of selected gene expression levels weighted by their corresponding regression coefficients derived from the LASSO Cox model.

### Construction and validation of TPFR (tumor-progression fibroblast cells riskscore) model

The TPFR prognostic model was constructed using the four-gene signature (*FOXA1, TBX3, LRIG1,* and *RNF11*) identified from LASSO Cox regression analysis. Risk scores were calculated using the formula: TPFR = Σ(coefficient × expression level) for each gene, where coefficients were derived from the Cox proportional hazards model implemented via the rms package in R. The TCGA-BLCA cohort (n = 359) served as the training dataset, while the E-MTAB-4321 dataset (n = 476) was employed as an independent external validation cohort. Patients in each dataset were stratified into high-risk and low-risk groups based on the median TPFR score.

Time-dependent ROC analysis was performed using the timeROC package to assess the predictive accuracy of the TPFR model at 1-, 3-, and 5-year time points. AUC values and their 95% confidence intervals were calculated using 10-fold cross-validation with marginal weighting for censoring distribution estimation.

### Immune infiltration analysis

ESTIMATE algorithm calculated stromal, immune, and ESTIMATE scores (Yoshihara et al., 2013). CIBERSORT (Newman et al., 2015) deconvoluted 22 immune cell subtypes using LM22 signature matrix with 1,000 permutations on TPM-normalized data. MCPcounter (Becht et al., 2016) quantified absolute abundance of eight immune and two stromal cell populations. Differences between CAF cluster were assessed by Wilcoxon rank-sum test. Correlations with risk scores used Pearson correlation (*P* < 0.05).

### Cell culture

Human embryonic kidney cell line HEK293 and human bladder cancer cell lines T24 and 5637 were obtained from the Cell Bank of Type Culture Collection of Chinese Academy of Sciences (Shanghai, China; http://www.cellbank.org.cn/). HEK293 cells were maintained in high-glucose DMEM supplemented with 10% fetal bovine serum (FBS) and 1% penicillin-streptomycin. T24 and 5637 cells were cultured in RPMI-1640 medium containing 10% FBS and 1% penicillin-streptomycin. All cell lines were incubated at 37 °C in a humidified atmosphere with 5% CO_2_ (Thermo Scientific, Forma 3111). Cells were passaged every 2 days at 70%–80% confluence using 0.25% trypsin-EDTA (Gibco, 25,200,056) for detachment. All experiments were performed using cells within passage 20.

### Lentivirus production and infection

Short hairpin RNA (shRNA) sequences specifically targeting human *RNF11* were synthesized by Tsingke Biological Technology (Beijing, China). The sequences were as follows: sh*RNF11*-1: 5′-GTGATCTGTATGATGGACTTT-3'; sh*RNF11*-2: 5′-GCTCAAAGAATAGGTCTTATA-3'; sh*RNF11*-3: 5′-GGATGACATCTCCCTGCTTCA-3'.

Lentiviral particles were produced using the calcium phosphate transfection method. Briefly, HEK293 cells were seeded in 6-well plates 1 day prior to transfection to achieve approximately 90% confluency on the day of transfection. Calcium phosphate transfection was performed using shRNA plasmid, packaging plasmid psPAX2, and envelope plasmid pMD2.G. After 24 h, the medium was replaced with 2 mL of fresh culture medium (DMEM, 10% FBS and 1% penicillin–streptomycin). Viral supernatants were harvested 48 h post-transfection and filtered through a 0.45 μm membrane to remove cell debris. Target cells were infected with lentiviral particles in the presence of 2 mg/mL polybrene for 6–8 h, and stably transduced cells were selected with puromycin.

### RNA extraction and qPCR

Cells (2 × 10^6^) from T24 and 5637 cell lines were harvested by centrifugation at 1,500 rpm for 5 min, followed by supernatant removal. After washing with 500 μL PBS (1,500 rpm, 5 min), 1 mL Trizol was added for cell lysis with thorough mixing at room temperature for 5 min. Subsequently, 200 μL chloroform was added with vigorous shaking for 30 s (mixing uniformly), followed by 20 min incubation on ice for phase separation. After centrifugation at 12,000 rpm, 4 °C for 20 min, 360 μL of the upper aqueous phase containing RNA was transferred to RNase-free EP tubes. An equal volume of isopropanol (360 μL) was added, inverted 5 times for mixing, and incubated at −80 °C for 2 h. Following centrifugation at 12,000 rpm, 4 °C for 10 min, the RNA pellet was visible and the supernatant was discarded. The pellet was washed with 1 mL of 75% DEPC-treated ethanol (DEPC:ethanol = 1:3), inverted 5–8 times, and centrifuged at 7,500 rpm, 4 °C for 8 min. After complete removal of supernatant, 40 μL DEPC water was added to dissolve the RNA. RNA concentration and purity were determined using NanoDrop spectrophotometer.

Reverse transcription was performed using HiScript II Q RT SuperMix for qPCR kit (Vazyme, R223-01). RNA concentration was adjusted, and 1 μg total RNA was used for reverse transcription. Quantitative PCR was performed using ChamQ Universal SYBR qPCR Master Mix (Vazyme, Q711-02). Primers are listed in Table 2. Gene expression levels were normalized by ACTB rRNA.

**TABLE 2:** qPCR Primer.

Gene	Primer
*RNF11* F	5′- AGAGGAACAAATTAGGATAGCTCA -3′
*RNF11* R	5′- TCGAATTGGGTCCCCATAAAC -3′
*ACTB* F	5′- GCCTCGCCTTTGCCGAT -3′
*ACTB* R	5′- CGCGGCGATATCATCATCC- 3′

### RNA sequencing

#### Cell handling, RNA preparation, and outsourcing

Human bladder cancer cell line 5637 was used to generate a stable *RNF11*-knockdown line (sh*RNF11*-1) and a matched control line (Ctrl), with n = 3 biological replicates per group (6 libraries in total; sample IDs: seq5-A-1/-2/-3 and seq5-Ctrl-1/-2/-3). Approximately 2.5 × 10^6^ cells per sample were lysed in 1 mL TRIzol, snap-frozen on dry ice, stored at −80 °C, and shipped to Berry Genomics Co., Ltd. (Beijing, China) for RNA-seq library preparation, sequencing, and bioinformatics.

#### Library preparation and sequencing

Poly(A)-selected non-strand-specific mRNA-seq libraries were constructed following the vendor’s standard workflow (fragmentation, first- and second-strand cDNA synthesis, end-repair/adapter ligation, PCR amplification, and QC), pooled equimolarly, and sequenced on an Illumina platform (PE150). Total output amounted to 50.41 Gb clean bases (mean 8.4 Gb per sample), with Q30 ≥ 93.97%; the mean rRNA fraction was 0.24%.

#### Read filtering and rRNA depletion

Adapter and low-quality bases were removed according to vendor criteria (e.g., discarding reads with ≥20% bases at Phred ≤5); remaining reads were aligned to the SILVA database with Bowtie2 to exclude rRNA.

#### Reference genome, alignment, and quantification

Clean reads were aligned to GRCh38.p14 (RefSeq build GCF_000001405.40) with HISAT2 using the corresponding GTF annotation. Reported overall mapping rates were 96.32% (minimum)/96.47% (average), with an average 69.8% of reads mapping to CDS regions. Gene-level counts were generated with featureCounts (exonic features aggregated to gene_id). Reference genome: https://ftp.ncbi.nlm.nih.gov/genomes/refseq/vertebrate_mammalian/Homo_sapiens/reference/GCF_000001405.40_GRCh38.p14/GCF_000001405.40_GRCh38.p14_genomic.fna.gz (NCBI RefSeq).

#### Normalization and differential expression

Differential expression was assessed with edgeR using the thresholds |log_2_FC| > 0.6 and FDR <0.05, q < 0.05 after library-size normalization.

#### RNA quality

RNA purity/integrity were evaluated by NanoDrop and Agilent 4,200; all samples passed vendor QC with RINe 9.7–10 and were graded Level I (eligible for library construction).

#### CCK-8 cell proliferation assay

T24 and 5637 cells were seeded in 96-well plates at a density of 2,000 cells per well in triplicate. Cell viability was assessed at 24, 48, 72, 96, and 120 h post-seeding using the Cell Counting Kit-8 (CCK-8; Biosharp, China). At each time point, the culture medium was replaced with 100 μL of fresh medium containing 10% CCK-8 reagent. After incubation at 37 °C for 0.5, 1, or 2 h, absorbance was measured at 450 nm using a microplate reader. The optimal incubation time was determined based on the linear range of the absorbance readings. Growth curves were generated by plotting absorbance values against time, and data were presented as mean ± standard deviation from three independent experiments.

### Wound healing assay

T24 and 5637 cells were seeded into 6-well plates and cultured until reaching approximately 90% confluence. A linear wound was generated in the cell monolayer using a sterile 200 μL pipette tip. After washing 3 times with PBS, the cells were incubated in serum-free medium. Images of the same wound sites were captured at 0 h, 12 h, and 24 h. The migration rate at each time point was calculated using the following formula: Migration rate (%) = [1 – (wound area at indicated time point/wound area at 0 h)] × 100%. Wound areas were quantified using ImageJ software (NIH, USA). Quantitative analysis was performed for both 12 h and 24 h post-scratch to assess cell migration dynamics.

### Transwell invasion assay

Matrigel was thawed overnight at 4 °C and diluted 1:8 with serum-free medium on ice. The upper chambers of Transwell inserts (8 μm pore size, Corning) were coated with 60 μL of diluted Matrigel and incubated at 37 °C for 3 h to allow solidification. After rehydration with 100 μL serum-free medium for 30 min, 200 μL of cell suspension in serum-free RPMI-1640 medium was added to the upper chambers (T24: 1.5 × 10^5^ cells/mL; 5637: 8 × 10^5^ cells/mL). The lower chambers were filled with 500 μL of complete RPMI-1640 medium containing 10% FBS as chemoattractant. After 24 h of incubation at 37 °C, non-invaded cells and residual Matrigel were gently removed from the upper surface using cotton swabs. Invaded cells on the lower surface were fixed with 4% paraformaldehyde for 30 min, stained with 0.1% crystal violet solution for 10 min, washed gently with PBS, and air-dried. Five random fields per insert were photographed under an inverted microscope at ×10 magnification. The number of invaded cells was quantified using ImageJ software.

### Software and statistical analysis

All statistical analyses were performed using R statistical software (version 4.5.0, R Core Team, 2025). Data manipulation and visualization were conducted using tidyverse (v2.0.0), dplyr (v1.1.4), ggplot2 (v3.5.2), ggrepel (v0.9.6), and reshape2 (v1.4.4). Non-negative matrix factorization (NMF) clustering was performed using NMF (v0.28). Differential expression analysis was conducted using limma (v3.64.1) with the voom transformation for RNA-seq data normalization. Functional enrichment analyses were performed using clusterProfiler (v4.16.0) with gene annotation from org.Hs.eg.db (v3.21.0). GO and KEGG pathway enrichment were visualized using enrichplot (v1.28.2) and GOplot (v1.0.2). Survival analyses were conducted using survival (v3.8.3) and survminer (v0.5.0) packages. Kaplan-Meier survival curves were generated, and log-rank tests were performed to compare survival differences between groups. Cox proportional hazards regression models were constructed to identify prognostic factors. Feature selection was performed using LASSO regression via glmnet (v4.1.9), with optimal lambda determined by cross-validation. Backward stepwise selection was conducted using leaps (v3.2). The prognostic risk model was constructed and visualized using rms (v8.0.0) and ggrisk (v1.3). Time-dependent receiver operating characteristic (ROC) curves were generated using timeROC (v0.4) to evaluate model performance. Heatmaps were generated using pheatmap (v1.0.13) with color schemes from RColorBrewer (v1.1.3). Additional visualizations were created using ggpubr (v0.6.0), grid (v4.5.0), and scales (v1.4.1).

Data are presented as means ± SEM. Two-tailed unpaired *t*-test was used to compare the difference between two groups. One-way ANOVA followed by Tukey’s multiple comparisons test was used to compare differences between multiple groups. *P* values <0.05 were considered significant. The level of significance is indicated as *P < 0.05, **P < 0.01, and ***P < 0.001. Statistical analysis was performed using Prism software.

## Data Availability

The datasets supporting the conclusions of this article are available in the following repositories: TCGA-BLCA dataset is available in the UCSC Xena platform repository, http://xena.ucsc.edu/; E-MTAB-4321 dataset is available in the ArrayExpress repository, https://www.ebi.ac.uk/arrayexpress/; GSE267718 dataset is available in the GEO repository, https://www.ncbi.nlm.nih.gov/geo/query/acc.cgi?acc=GSE267718. Additional supporting data are included as supplementary files with this article.

## Glossary

BLCA: Bladder cancer
MIBC: Muscle-invasive bladder cancer
NMIBC: Non-muscle-invasive bladder cancer
TURBT: Transurethral resection of bladder tumor
CAFs: Cancer-associated fibroblasts
iCAFs: Inflammatory cancer-associated fibroblasts
myCAFs: Myofibroblastic cancer-associated fibroblasts
ICI: Immune checkpoint inhibitor
PD-L1: Programmed death-ligand 1
scRNA-seq: Single-cell RNA sequencing
RSS: Residual sum of squares
DEGs: Differentially expressed genes
NMF: Non-negative matrix factorization
PCA: Principal component analysis
TME: Tumor microenvironment
OS: Overall survival
OSS: Overall survival status
DFS: Disease-free survival
DSS: Disease-specific survival
ECM: Extracellular matrix
EGFR: Epidermal growth factor receptor
ESTIMATE: Estimation of STromal and Immune cells in MAlignant Tumours using Expression data
CIBERSORT: Cell-type Identification By Estimating Relative Subsets Of RNA Transcripts
FDR: False discovery rate
AIC: Akaike information criterion
LASSO: Least Absolute Shrinkage and Selection Operator
ROC: Receiver Operating Characteristic
TPFR: Tumor-Progressing Fibroblast Riskscore
TNF: Tumor necrosis factor
MAPK: Mitogen-activated protein kinase
NES: Normalized enrichment score
NF-κB: Nuclear factor-κB
PTM: Post-translational modification
CCK-8: Cell Counting Kit-8
qPCR: Quantitative polymerase chain reaction
